# Factors influencing physiotherapy decisions between restorative and compensatory gait rehabilitation: an Italian multicenter study

**DOI:** 10.3389/fneur.2024.1368973

**Published:** 2024-05-24

**Authors:** Fabiola G. Mestanza Mattos, Thomas Bowman, Francesca Marazzini, Silvia Salvalaggio, Cristina Allera Longo, Serena Bocini, Viviana Bonci, Francesco G. Materazzi, Elisa Pelosin, Martina Putzolu, Andrea Turolla, Susanna Mezzarobba, Davide Cattaneo

**Affiliations:** ^1^Department of Pathophysiology and Transplantation, Università degli Studi di Milano, Milan, Italy; ^2^IRCCS Fondazione Don Carlo Gnocchi, Milan, Italy; ^3^AIAS di Milano Onlus, Milan, Italy; ^4^Laboratory of Computational Neuroimaging, IRCCS San Camillo Hospital, Venice, Italy; ^5^Padova Neuroscience Center, Università degli Studi di Padova, Padua, Italy; ^6^Department of Rehabilitation, San Carlo Borromeo Hospital, Milan, Italy; ^7^Division of Physical and Rehabilitation Medicine, Fondazione Opera San Camillo, Presidio di Torino, Italy; ^8^Department of Neurological Sciences, Neurorehabilitation Clinic, AOU delle Marche, Ancona, Italy; ^9^Montecatone Rehabilitation Institute, Imola (BO), Italy; ^10^Department of Biotechnological and Applied Clinical Sciences (DISCAB), University of L’Aquila, L'Aquila, Italy; ^11^Department of Neuroscience, Rehabilitation, Ophthalmology, Genetics, Maternal and Child Health, University of Genoa, Genoa, Italy; ^12^IRCCS Ospedale Policlinico San Martino, IRCCS, Genoa, Italy; ^13^Laboratory Department of Experimental Medicine (DIMES), Section of Human Physiology, University of Genoa, Genoa, Italy; ^14^Department of Biomedical and Neuromotor Sciences (DIBINEM), Alma Mater Studiorum - Università di Bologna, Bologna, Italy; ^15^Unit of Occupational Medicine, IRCCS Azienda Ospedaliero-Universitaria di Bologna, Bologna, Italy; ^16^Azienda Sanitaria Universitaria Giuliano Isontina (ASUGI), Trieste, Italy

**Keywords:** neurological rehabilitation, physical therapy modalities, stroke, multiple sclerosis, Parkinson’s disease

## Abstract

**Background and purpose:**

This study aimed to investigate the factors that influence physiotherapists’ decision in choosing restorative or compensatory rehabilitation during gait training in people with neurological disorders (PwNDs) and the different treatments used in the approaches.

**Methods:**

This cross-sectional analysis used the baseline data from an observational cohort study. We analyzed data from 83 PwNDs (65 people after stroke, 5 with multiple sclerosis, and 13 with Parkinson’s disease) who underwent at least 10 sessions of physiotherapy (PT) focusing on gait function. Performance was quantified using the modified Dynamic Gait Index (MDGI), three impairment domains of Fugl–Meyer Assessment for lower extremity (mFM-LL), Activities-specific Balance Confidence (ABC), modified Barthel Index (mBI), Mini-Mental State Examination (MMSE), and Motivational Index (MI). Forty-three physiotherapists completed a treatment report form categorizing the rehabilitation approach and specifying treatments used (e.g., resistance training and proprioceptive exercises).

**Results:**

Fifty-six subjects underwent restorative rehabilitation approach. The univariate predictors of restorative approach were being in the subacute phase with a disease onset of less than 180 days, (odds ratio [95%CI]; 3.27[1.19–9.24]), mFM-LL (1.25[1.11–1.44]), MMSE (0.85[0.67–1.00]), and number of sessions (1.03[1–1.01]). The backward stepwise analysis revealed an association between restorative and subacute phase (36.32[4.11–545.50]), mFM-LL (3.11[1.55–9.73]), mBI (1.79[1.08–3.77]), MMSE (0.46[0.25–0.71]), and the interaction between mFM-LL and mBI (0.99[0.98–1.00]). No statistically significant association between treatments used and approach was found (*p* = 0.46).

**Discussion and conclusion:**

The restorative approach was more commonly used to improve gait. The main variables associated with this approach were: being in the subacute phase of the disease, a low level of impairment, and a high level of functional independence at baseline. However, few differences were found between the treatments used for the restorative or compensatory approaches, as similar PT treatments were used for both.

## Introduction

People with neurological disorders (PwNDs) typically present limitations in performing functional tasks (1, 2). Therefore, a major focus of neurological rehabilitation is to maximize functional motor abilities, such as walking (3). It has been postulated that functional improvements occur due to a combination of two phenomena: (1) motor recovery via the relearning of premorbid motor skills and (2) motor compensation via learning new movement strategies (4).

Although both motor recovery and compensation can lead to functional improvements, the decision between the two approaches is not trivial. When motor recovery is prioritized, more emphasis is placed on movement quality, providing more feedback on movement performance, leading to the relearning of skilled movements. Conversely, when compensation is prioritized, physiotherapists train people to use assistive devices or other motor strategies. As a result, less attention is paid to the quality of the movement, and this could lead to different rehabilitation outcomes, as suggested by Krakauer (5).

The factors leading to the selection of motor recovery or compensation are unknown. The clinical decision-making process may be influenced by evidence-based practice recommendations and implicit, subjective theories based on clinical experience and contextual factors (6, 7). The physiotherapist’s expectation of good functional improvement may lead to the adoption of a restorative rather than a compensatory approach. Factors influencing the outcome of motor rehabilitation vary. These include people after stroke age, the severity of walking disabilities, and balance impairment at baseline (8, 9). For people with multiple sclerosis (MS), the impact of balance impairment, disease severity, and disease progression on gait rehabilitation outcomes is still under debate (10–12). In people with Parkinson’s disease (PD), age, motor and cognitive impairment, and disease severity were factors associated with gait improvements (13).

To date, no studies have examined the relationship between these factors and the clinical decision-making process in everyday clinical practice. Our hypothesis is that the restorative approach is chosen when a larger number of sessions are available and clinical characteristics suggest a good clinical outcome. The aim of this study was to investigate the factors influencing the choice between rehabilitation approaches. The secondary aim of this study was to investigate whether restorative or compensatory approaches consisted of different physiotherapy (PT) treatments in everyday clinical practice.

## Methods

### Study design

Data for this cross-sectional study were collected in a larger multicenter longitudinal prospective cohort study investigating the contents of neurological PT (see Supplementary Figure 1) (3). The multicenter network involved a total of nine facilities: four research/university hospitals, two general hospitals, and three rehabilitation centers. All centers provided multidisciplinary rehabilitation, including both outpatient and inpatient services.

The study was registered on clinicaltrial.gov (ID: NCT04386863) and was approved by the Ethical Committee of [redacted]. Recruitment began in June 2018 and ended in July 2021. The study was conducted in agreement with the Declaration of Helsinki, and all participants provided written informed consent.

#### Participants

A convenience sample was recruited for the study from a variety of rehabilitation facilities in order to minimize the potential for selection bias. All the eligible subjects were recruited consecutively. Participants were individuals after stroke and those with MS or PD who met the following inclusion criteria: age > 18 years and had received at least 10 20- to 50-min PT sessions aimed at improving walking function. Only subjects who were unable to understand the aims of the study and provide informed consent were excluded.

#### Experimental procedures

The recruited subjects followed rehabilitation programs set by a multidisciplinary team, including healthcare professionals and physicians. All participants underwent PT sessions foreseen by the National Healthcare System and were clinically assessed before the treatment. All clinical evaluations were performed by an experienced clinical researcher not involved in the PT sessions. For each participant, the physiotherapist who administered the treatment completed a treatment report form at the end of the rehabilitation program. In the case of a physiotherapist treating more than one subject, a form for each subject being treated was completed.

#### Clinical assessment

Demographic information, such as sex, age, and disease onset, was collected, along with clinical outcomes measured at the beginning of the rehabilitation program to characterize participants. Subjects with a disease onset of less than 180 days were considered subacute. Thus, subjects with MS and PD were considered all chronic. The modified Dynamic Gait Index (MDGI) is a clinical scale that was used to assess balance performances in dynamic tasks (the best score is 64 points), while the Activities-specific Balance Confidence (ABC) is a 16-item questionnaire used to rate individuals’ self-perceived balance confidence during various activities of daily living (100 points meaning complete confidence) (14–16).

Alterations of tactile and proprioceptive sensation, passive joint motion, and joint pain in the lower extremities were assessed using several items from the Fugl–Meyer Assessment for Lower Limb (mFM-LL) to use a reproducible and known assessment procedure. We selected these domains since they are useful for evaluating lower limb impairments not rated by functional tests (17). Scores for the sensation ranged from 0 to 10, scores of the passive joint motion ranged from 0 to 32, and finally, scores for the joint pain ranged from 0 to 20, with higher scores indicating lower levels of impairment. The sum of the scores of the three domains was calculated to provide a summary of sensory-motor impairments. Both lower limbs were assessed, but only the score from the most affected limb was considered for this study.

Finally, we used the modified Barthel index (mBI) to quantify functional independence (100 points meaning complete independence) and the Mini-Mental State Examination (MMSE) to assess cognitive functions (best score of 30 points) (18, 19). The Motivational Index (MI) was used to investigate participants’ motivation and engagement within the rehabilitation process (score from 7 to 35 points) (20).

#### Physiotherapist treatment report form

Through a treatment report form, we asked physiotherapists to identify the number of PT sessions provided, the approach used (by checkbox), and the treatments used during the PT program (see Supplementary Figure 2). The physiotherapists involved had to define the approach used during the rehabilitation program (restorative or compensatory) based on the purpose of the intervention provided, i.e., improving walking function. The definitions of restorative and compensatory approaches were provided to the physiotherapists. Restorative approach was defined as rehabilitation aimed at restoring premorbid walking patterns, while compensatory approach was defined as rehabilitation aimed at promoting new motor patterns to walk (4).

### Statistical analysis

Each PwND was categorized as receiving a restorative or compensatory approach according to the physiotherapist’s classification. Then, the sample of PwNDs was split into two subgroups, one treated with a restorative approach and the other with a compensatory approach. Descriptive statistics consisted of group means and standard deviations of demographics and clinical outcomes.

Bivariate and multivariate logistic regression models were conducted using the approach (compensatory = 0; restorative = 1) as dependent variable and clinical measures as independent variables. All variables were used as predictors of the approach except for sex, which was considered a confounder. A bivariate analysis was performed using one independent predictor at a time. To improve stability and control for variance inflation, determinants were removed if collinearity was of concern in a model without interactions (variance inflation factor (VIF) > 5). All variables showing at least a weak association with the dependent variable (*p* < 0.1) were entered into a multivariate model. Since the interaction between variables measuring impairment and independence in activities of daily living was associated with the dependent variable, it was also included in the subsequent analyses. Finally, a backward stepwise logistic regression was used to identify the most parsimonious model using the stepAIC procedure from the MASS R library. Plots of residuals were used to check for the homogeneity of variance, and qq plots were used to check for normality of distributions of residuals, while Cook’s distance provided an indication of the presence of influential observations.

To verify the consistency of results, we carried out a sensitivity analysis running the same model on a subpopulation of people after stroke in the subacute phase only (onset<180 days), which was the largest subsample in our study.

To check the association between approaches and treatments provided during rehabilitation, we used contingency tables and an overall chi-squared test, removing treatments with less than 10 occurrences.

A significance level of *p* < 0.05 was set for all tests, and missing data were not imputed. All analyses were performed using R Statistical Software version 4.2.2 (R Core Team 2022).

## Results

### Participants characteristics

Data from 83 participants (65 people after stroke, 5 people with MS, and 13 people with PD) were analyzed, and demographic and clinical characteristics for the restorative and compensatory groups are shown in Table 1. A total of 43 physiotherapists were involved and completed the treatment form.

**Table 1:** Demographic and clinical characteristics of the restorative and compensatory groups.

Characteristic	Overall *N* = 83	C *N* = 27	R *N* = 56	OR	95%CI	*p*-value
Sex (#, F)	34 / 83 (41%)	12 / 27 (44%)	22 / 56 (39%)	1.24	0.48–3.17	0.67
Age (years)	68.41 (11.12)	68.44 (10.13)	68.39 (11.66)	1.00	0.96–1.04	0.98
Setting (#, Inpatients)	62/83 (75%)	17/27 (63%)	45/56 (80%)	0.42	0.15–1.17	0.10
Subacute phase (#)	60 / 83 (72%)	15 / 27 (56%)	45 / 56 (80%)	3.27	1.19–9.24	**0.02**
Sessions (#)	24.02 (17.06)	18.70 (7.96)	26.59 (19.57)	1.04	1.00–1.08	**0.06**
MDGI (points)	22.95 (18.80)	24.46 (22.26)	22.26 (17.20)	0.99	0.97–1.02	0.64
ABC (points)	36.64 (26.96)	38.41 (27.80)	35.81 (26.78)	1.00	0.98–1.01	0.69
mFM-LL (points)	57.04 (5.61)	55.11 (4.88)	57.98 (5.74)	1.25	1.11–1.44	**<0.01**
mBI (points)	66.00 (25.21)	68.33 (27.39)	64.88 (24.27)	0.99	0.98–1.01	0.56
MMSE (points)	27.40 (2.98)	28.19 (2.14)	27.02 (3.26)	0.85	0.67–1.00	**0.09**
MI (points)	27.41 (3.31)	27.56 (2.59)	27.34 (3.62)	0.98	0.85–1.13	0.78

C, compensatory approach; R, restorative approach; OR, odd ratio (reference category: C), 95%CI, 95% confidence interval of OR; MDGI, modified Dynamic Gait Index; ABC, Activities-specific Balance Confidence; mFM-LL, modified Fugl–Meyer Lower limb; mBI, modified Barthel Index; MMSE, Mini-Mental State Examination; MI, motivational index.The bold values for associations with a *p*-value below 0.10.

### Factors identification

#### Whole sample

The univariate analyses revealed that subacute phase ((OR[95%CI] 3.28[1.19–9.24]), mFM-LL (1.25[1.11–1.44]), and MMSE 0.85[0.67–1.00]), and number of available sessions (1.04[1.00–1.08]) were associated with the restorative approach. These relationships are depicted in Supplementary Figure 3. Furthermore, the interaction between impairment and independence was also statistically associated with the restorative approach (0.99[0.98–1.00]).

**Figure 1:**
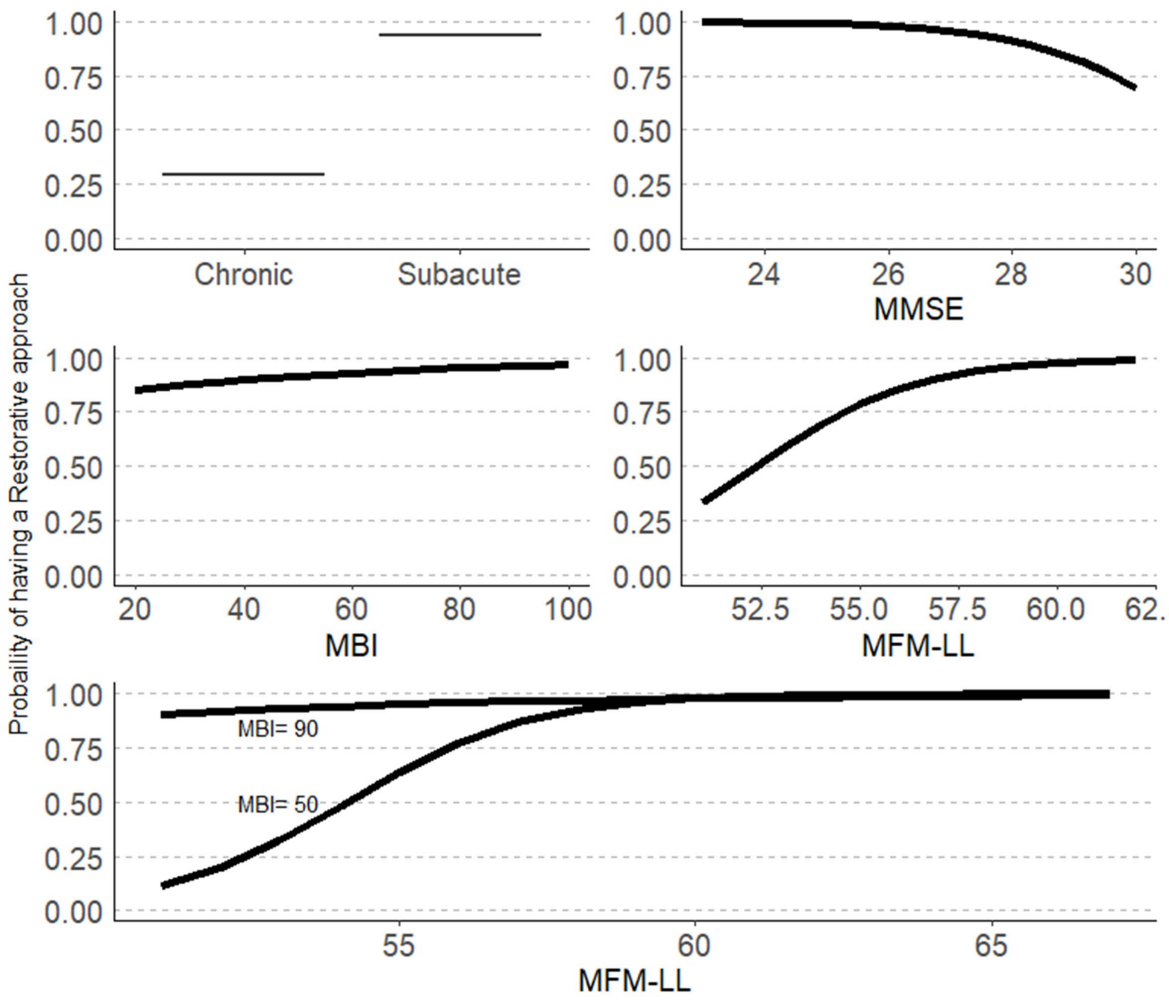
Probability of having a restorative approach based on stepwise logistic regression analyses. MMSE, Mini-Mental State Examination; mFM-LL, modified Fugl–Meyer Lower limb; mBI, modified Barthel Index.

Based on the results of the bivariate logistic regression analyses (see Table 1 and Supplementary Figure 3), we included five independent variables in the multivariate model: onset, mFM-LL, mBI, MMSE, and the number of sessions, along with the interaction between mFM-LL and mBI. The stepwise multivariate logistic regression result is presented in Table 2, with the associations represented in Figure 1.

**Table 2:** Stepwise logistic regression model including best clinical predictors of restorative or compensatory approach.

Clinical variables	OR	95%CI	*p*-value
Subacute phase	36.32	4.11–545.50	<0.01
mFM-LL	3.11	1.55–9.73	0.01
mBI	1.79	1.08–3.77	0.06
MMSE	0.46	0.25–0.71	<0.01
mFM-LL: mBI	0.99	0.98–1.00	0.07

AIC = 58.11; R^2^ McFadden = 0.49. OR, odds ratio; 95%CI, 95% confidence interval; mFM-LL, modified Fugl–Meyer Lower limb; mBI, modified Barthel Index; MMSE, Mini-Mental State Examination.

Being a subject after stroke during the subacute phase (onset <180 days), not severely impaired, and independent in daily life activities were associated with a higher probability of receiving a restorative rehabilitation approach. Cognitive status was significantly associated with a restorative approach, with more impaired subjects having a higher probability of receiving a restorative approach. In addition, the lower panel of Figure 1 shows the interaction between impairment and independence, where the probability of receiving a compensatory approach is high only in dependent participants (lower mBI scores) showing high levels of sensory-motor impairments (lower mFM-LL scores).

#### Subacute sample

We performed a stepwise multivariate logistic regression (Table 3) exclusively on the subacute population (onset<180 days). The multivariate model included independent variables associated with the restorative approach in the univariate analyses.

**Table 3:** Stepwise logistic regression model including best clinical predictors of restorative or compensatory approach on the subacute population.

Clinical variables	OR	95%CI	*p* value
mFM-LL	4.67	1.69–29.21	0.03
mBI	2.77	1.17–10.72	0.07
MMSE	0.43	0.13–0.90	0.08
mFM-LL:mBI	0.98	0.96–1.00	0.08

AIC = 38.03; R^2^ McFadden = 0.51. OR, odds ratio; 95%CI, 95% confidence interval; mFM-LL, modified Fugl–Meyer Lower limb; mBI, modified Barthel Index; MMSE, Mini-Mental State Examination.

As expected, the *p*-value increased due to the reduction in sample size. However, lower limb sensory-motor impairment was significantly associated with a higher probability of receiving the restorative approach, and the other predictors were close to statistical significance.

### Contents of different rehabilitation approaches

**Figure 2:**
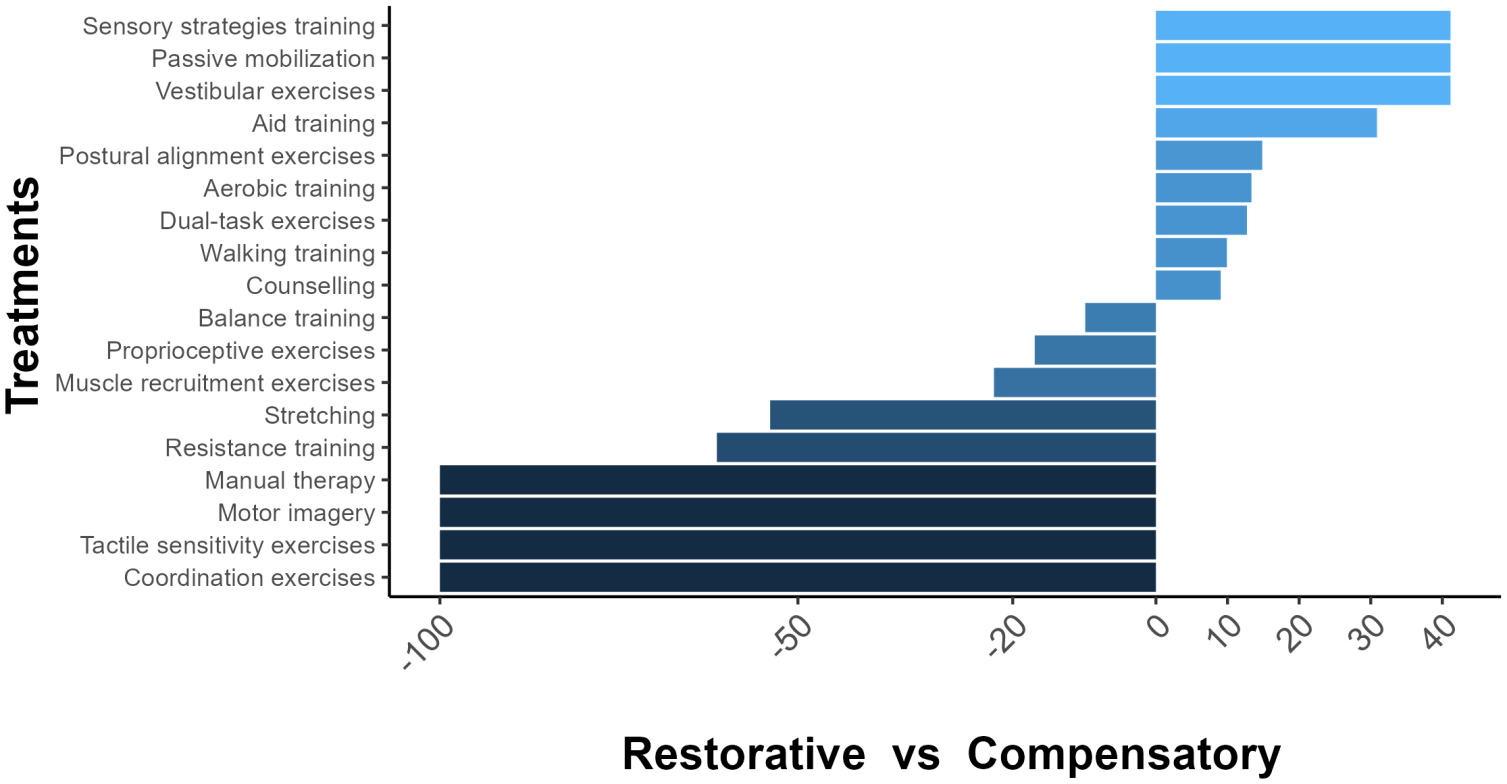
Percentages of treatment used in restorative and compensatory rehabilitation approaches. −100 = The treatment was administered for the restorative approach only. 100 = The treatment was administered for the compensatory approach only. 0 = The treatment was administered for both approaches.

Walking training, balance training, and proprioceptive exercises were the most prevalent treatments to improve walking in PwNDs (Supplementary Figure 4). Figure 2 shows the percentages of treatment used for restorative or compensatory approaches. Despite the differences observed across interventions, no statistically significant association between approach and treatment was found using the chi-square test (*p* = 0.46).

## Discussion

This unique observational study investigated the factors associated with the selection of restorative or compensatory approaches in PT clinical practice. We identified four main variables associated with the restorative rehabilitation approach: being in the subacute phase of the disease, having a low level of impairment at baseline with a high level of functional independence, and having lower cognitive performance.

The physiotherapists interviewed were all able to define their intervention as restorative or compensatory, indicating that the PT theoretical frameworks are well recognized by clinicians (4, 21). Both approaches were used to treat PwNDs, but the restorative approach was used more often than the compensatory approach in everyday clinical practice.

Our results suggest that specific clinical characteristics are associated with physiotherapists’ choice of approach. The factor that was most strongly associated with the restorative approach was the onset, with subacute subjects being 36 times more likely to receive restorative rehabilitation compared to chronic ones. The findings of this study support the *a priori* hypothesis that a physiotherapist’s decision-making process was influenced by the expectation of a good clinical outcome, and thus a restorative approach was more likely chosen.

Spontaneous neurological recovery is by far the largest contributor to behavioral restitution, and it is known that this phenomenon occurs during the first few weeks after stroke (subacute phase) (22, 23). Similarly, severe impairment was positively associated with worse clinical outcomes in longitudinal studies predicting walking recovery, and this association was also present when considering independence (mBI). Interestingly, we found a complex interaction between impairment and functional impendence. Severely impaired subjects were likely treated with a compensatory approach unless their independence level was high.

In other studies, preserved cognitive status was a predictor of good clinical outcome, which contrasts with our findings (24, 25). However, our results should be treated with caution, as the mean MMSE score was close to the highest score on the scale, meaning that the cognitive impairments measured by this test were rare in our sample. The MMSE has been widely used as a screening tool for dementia, but it has been criticized for not being sensitive enough to cognitive impairments specific to these populations, suggesting that other validated tests should be used in this population (26–28). Moreover, average MMSE scores were similar for subjects treated with a restorative and compensatory approach, and the association found between MMSE scores and the restorative approach was unexpected.

In contrast with our hypothesis, other variables associated with good clinical outcomes were not associated with the restorative approach. We anticipated that the age of individuals with PwNDs would influence the physiotherapist approach, with older subjects more likely to receive the compensatory approach. This expectation is based on previous reports indicating that older age is associated with poorer rehabilitation outcomes in individuals after stroke and in those with PD (13, 29). Similarly, balance performance and subjects’ motivation for PT were not considered in the choice of rehabilitation approach, although both factors have been shown to be associated with functional outcomes at the end of rehabilitation (9, 11, 12, 30).

In addition, we expected that a greater number of available sessions would influence the physiotherapist’s decision to select a restorative approach, as the time spent in rehabilitation should result in an increased therapy dosage (31, 32). The lack of association between the number of sessions and the restorative approach may be due to a lack of strong evidence supporting a dose–response effect in PwNDs (33–35).

Walking training, balance training, and proprioceptive exercises were frequently used to improve walking function in accordance with international guidelines for stroke, MS, and PD (35–37). It should be noted that a comprehensive data collection on the content of gait rehabilitation was beyond the scope of this study, as this topic has been investigated elsewhere (3). The treatments described in this study are limited to the everyday clinical practice of the participating rehabilitation centers. Other common PT approaches, such as dance or aqua therapy (38, 39), were not investigated.

Our findings indicated that certain interventions were exclusive to the restorative approach, such as manual therapy and motor imagery. However, most treatments used were similar for both approaches, resulting in a non-statistically significant difference. A more detailed description of the PT treatments is needed to understand the specifics between the restorative and compensatory approaches.

The study has certain limitations. The definitions of compensation and restitution in PwNDs, by Levin et al. (4), were used in this investigation. However, different interpretations of these concepts among physiotherapists represent a potential for subjective bias in their responses. Furthermore, we asked physiotherapists to classify their interventions on PwNDs at the conclusion of the PT program. Some interventions may have started with a restorative approach and then shifted to a compensatory approach or vice versa from session to session, depending on the needs of PwNDs. It was not possible to collect this type of information using our physiotherapist treatment report form.

No information was collected on the composition of the multidisciplinary rehabilitation team.

Finally, we did not consider other variables that have already been identified as predictors of good rehabilitation outcomes, such as the early presence of lower limb spasticity and muscle strength (40) or disease severity (12, 13, 41).

## Conclusion

In this sample, a restorative approach was often used in neurological rehabilitation. The main factor that influenced the choice of a restorative approach was predicted clinical improvement.

## Data availability statement

The raw data supporting the conclusions of this article will be made available by the authors, without undue reservation.

## Ethics statement

The studies involving humans were approved by Ethics Committee of the Don Gnocchi Foundation. The studies were conducted in accordance with the local legislation and institutional requirements. The participants provided their written informed consent to participate in this study. Written informed consent was obtained from the individual(s) for the publication of any potentially identifiable images or data included in this article.

## Author contributions

FMes: Conceptualization, Data curation, Formal analysis, Methodology, Project administration, Writing – original draft, Writing – review & editing. TB: Data curation, Formal analysis, Investigation, Methodology, Project administration, Writing – original draft, Writing – review & editing. FMar: Data curation, Investigation, Methodology, Writing – original draft, Writing – review & editing. SS: Data curation, Investigation, Writing – original draft, Writing – review & editing. CA: Data curation, Investigation, Writing – original draft, Writing – review & editing. SB: Data curation, Investigation, Writing – original draft, Writing – review & editing. VB: Data curation, Investigation, Writing – original draft, Writing – review & editing. FMat: Data curation, Investigation, Writing – original draft, Writing – review & editing. EP: Data curation, Investigation, Writing – original draft, Writing – review & editing. MP: Data curation, Investigation, Writing – original draft, Writing – review & editing. AT: Data curation, Investigation, Methodology, Writing – original draft, Writing – review & editing. SM: Funding acquisition, Investigation, Methodology, Supervision, Writing – original draft, Writing – review & editing. DC: Conceptualization, Formal analysis, Funding acquisition, Investigation, Methodology, Supervision, Writing – original draft, Writing – review & editing.
