# Supplementary material for: Factors influencing physiotherapy decisions between restorative and compensatory gait rehabilitation: an Italian multicenter study
Source: Front Neurol. 2024 May 24;15:1368973. doi: 10.3389/fneur.2024.1368973 (PMC11157038; doi:10.3389/fneur.2024.1368973)
Supplement: Supplementary file 1 [file Data_Sheet_1.PDF]

Supplementary Material

Fig. S1. Flow diagram of analysed subjects

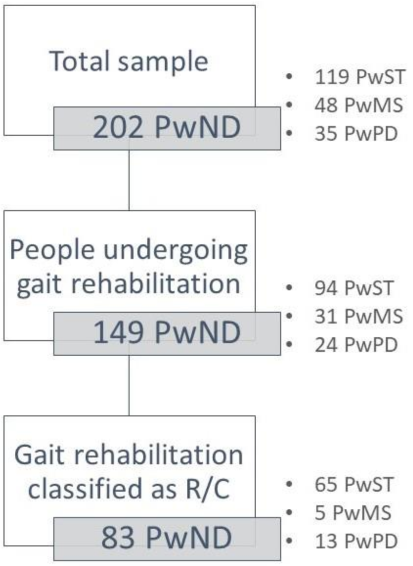

PwND: People with neurological disorders, PwST: people after stroke, PwMS: people with multiple sclerosis, PwPD people with Parkinson's disease, R: restorative approach, C: compensatory approach.

Fig. S2. Treatment report form administered to physiotherapists.

PT INTERVENTION FORM

Date_______________

Therapeutic goals	Priority	Approach	Time
	(1,2,3)	R/C	%
**Functional - Activities**			sum of the 3 percentages MUST BE = 100%
Manipulation			
Walking			
Postural transitions			
Stair climbing			
ADL			
**Impairment**			
Pain			
Joint mobility			
Muscle strength			
Sensory functions			
Balance			
Trunk control			
Standing position			
Tertiary prevention			
Cardiorespiratory fitness			
Cognitive function			
Genitourinary functions			
**Participation**			
Outdoor mobility			
Quality of life			
**Other**			

LEGEND
**Preliminary remark:** Not all the possible therapeutic goals or treatments are already indicated in this form, please use the proper empty spaces to indicate what is not already included.
**Priority:** please indicate up to 3 main rehabilitative aims and give them a level of priority: **1(high), 2(medium), 3 (low).**
**Time %:** indicate the percentage of **time dedicated** to each selected goal, compared to total time of treatment.
**IN/OUT PATIENT:** indicate whether the setting of rehabilitative intervention is hospital-based **(IN)** or not **(OUT)**.
**Restorative approach (R):** rehabilitation's goal is to restore premorbid capacity of injured functions. **Compensatory approach(C):** rehabilitation's goal is to vicariate lost functions finding new strategies.

SUBJECT DATA	
**Name** ______________________________	
Sessions frequence: n° ______ times/week; Total number of sessions: _____	
Session lasting:___ min	**IN/OUT PATIENT:** ☐ IN ☐ OUT
Other Therapies:	☐Speech/swallow rehabilitation ☐Occupational Therapy ☐Neuropsychologist ☐Massage Therapy ☐___________________ (Other)

Interventions	Goal 1	Goal 2	Goal 3
	% time	% time	% time
Aid training			
Aerobic training			
Postural alignment exercises			
Constraint induced movement therapy			
Counselling			
Tactile sensitivity exercises			
Dual-task execises			
Manipulation-grasping exercises			
Proprioceptive exercises			
Vestibular exercises			
Motor imagery			
Limphatic drainage			
Passive mobilization			
Muscle recruitment exercises			
Resistance training			
Stretching			
Manual therapy			
Instrumental physical therapies (specify)____________________			
Walking training			
Balance training			
Sensory strategies training			
Myofascial release			

Note: Indicate upt to 5 interventions type for each goal. Specify the percentage time dedicated to each intervention. The sum must be 100% for each goal.

Fig. S3. Probability of having a R approach based on bivariate logistic regression analyses.

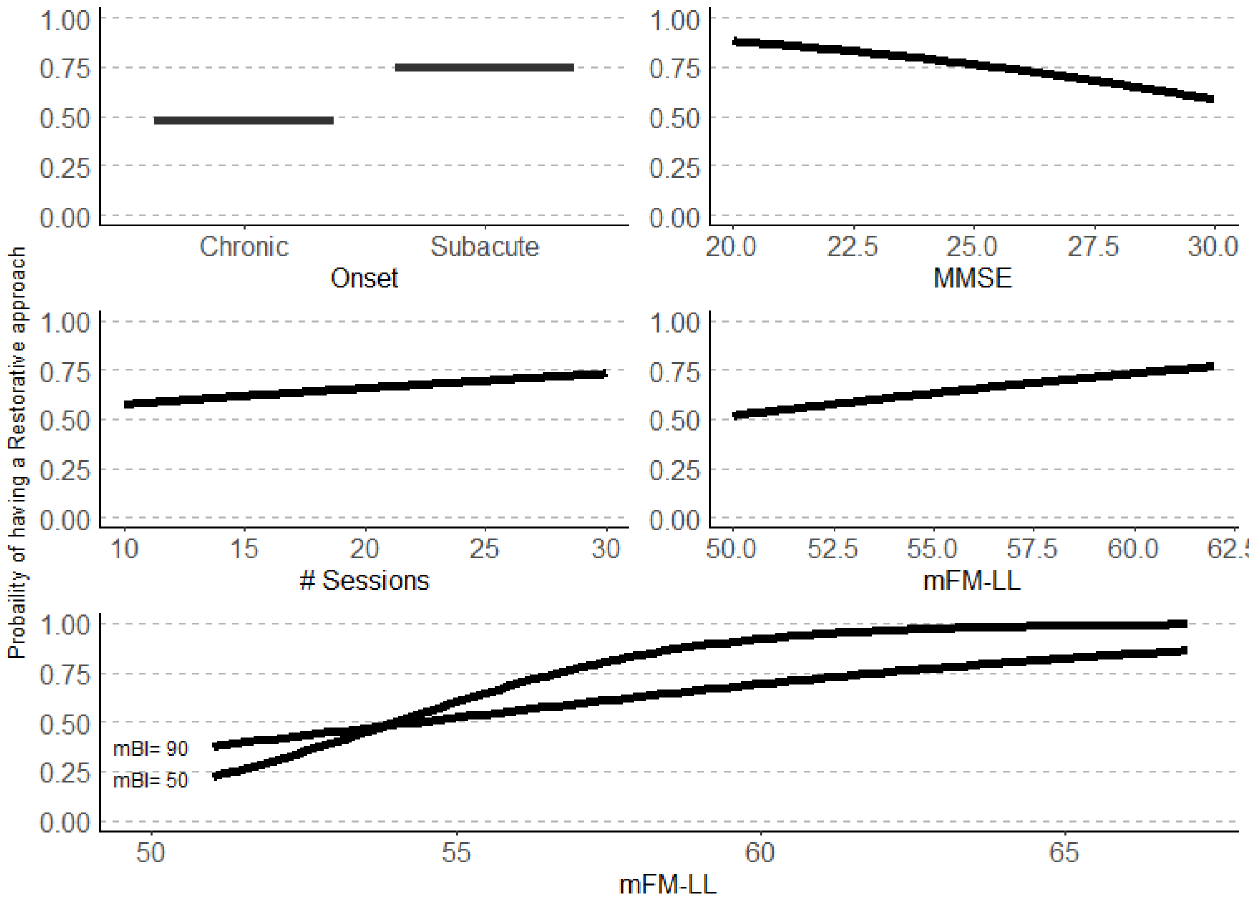

R: restorative, MMSE: Mini-Mental State Examination, mFM-LL: modified Fugl-Meyer Lower limb, mBI: modified Barthel Index.

Fig. S4. Interventions used to improve walking in PwND

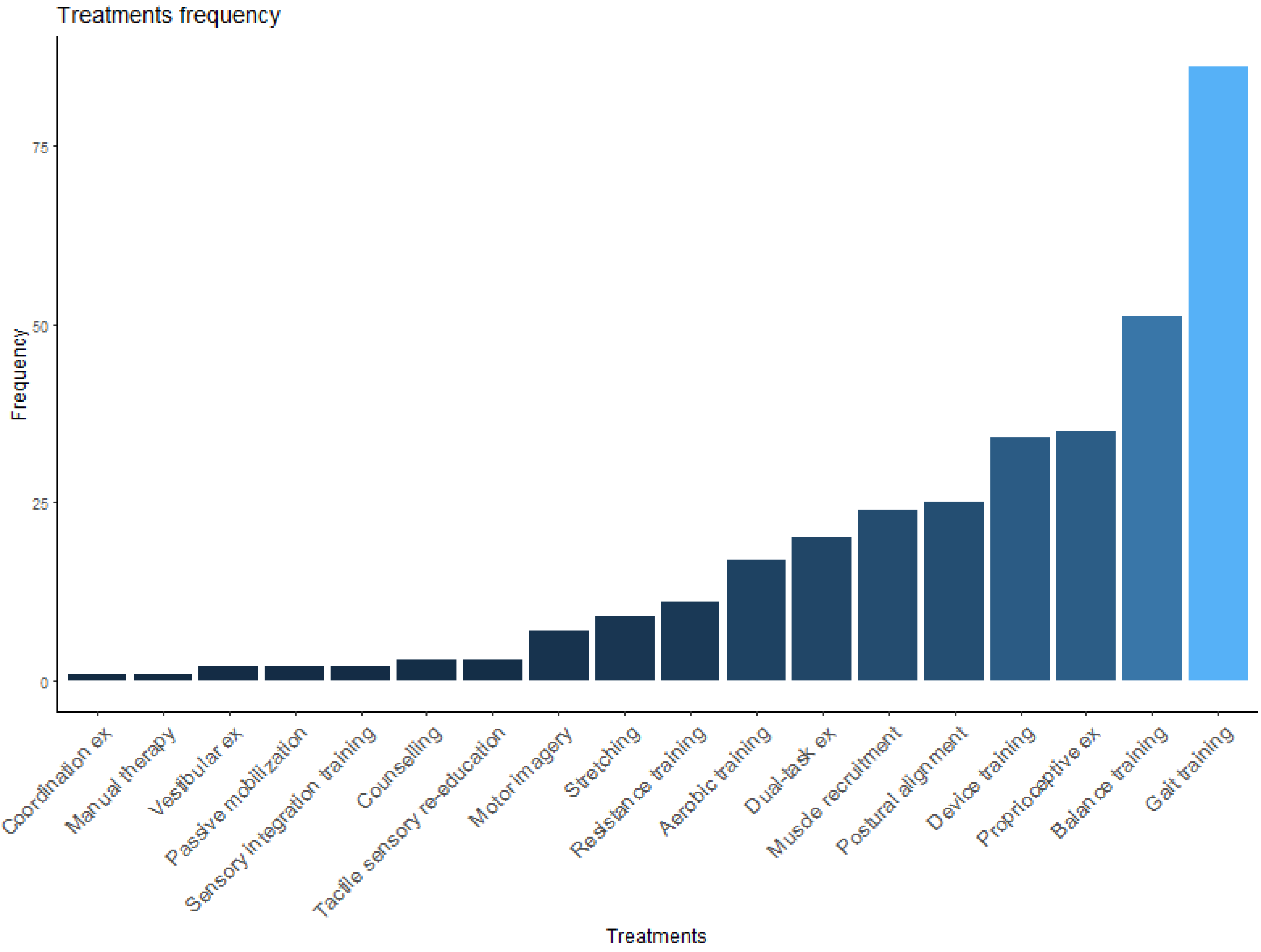

PwND: People with neurological disorders
